# A cyclopalladated complex interacts with mitochondrial membrane thiol-groups and induces the apoptotic intrinsic pathway in murine and cisplatin-resistant human tumor cells

**DOI:** 10.1186/1471-2407-11-296

**Published:** 2011-07-14

**Authors:** Fabiana A Serrano, Alisson L Matsuo, Priscila T Monteforte, Alexandre Bechara, Soraya S Smaili, Débora P Santana, Tiago Rodrigues, Felipe V Pereira, Luis S Silva, Joel Machado, Edson L Santos, João B Pesquero, Rafael M Martins, Luiz R Travassos, Antonio CF Caires, Elaine G Rodrigues

**Affiliations:** 1 Unidade de Oncologia Experimental, Departamento de Microbiologia, Imunologia e Parasitologia, Universidade Federal de São Paulo, São Paulo, Brazil; 2 Departamento de Farmacologia, Universidade Federal de São Paulo, São Paulo, Brazil; 3 Centro Interdisciplinar de Investigação Bioquímica, Universidade de Mogi das Cruzes, Mogi das Cruzes, São Paulo, Brazil; 4 Centro de Ciências Naturais e Humanas, Universidade Federal do ABC, São Paulo, Brazil; 5 Disciplina de Biologia Celular, Departamento de Microbiologia, Imunologia e Parasitologia, Universidade Federal de São Paulo, São Paulo, Brazil; 6 Departamento de Ciências Biológicas, Universidade Federal de São Paulo, São Paulo, Brazil; 7 Faculdade de Ciências Biológicas e Ambientais, Universidade Federal da Grande Dourados, Mato Grosso do Sul, Brazil; 8 Departamento de Biofísica, Universidade Federal de São Paulo, São Paulo, Brazil; 9 Disciplina de Parasitologia, Departamento de Microbiologia, Imunologia e Parasitologia, Unversidade Federal de São Paulo, São Paulo, Brazil

## Abstract

**Background:**

Systemic therapy for cancer metastatic lesions is difficult and generally renders a poor clinical response. Structural analogs of cisplatin, the most widely used synthetic metal complexes, show toxic side-effects and tumor cell resistance. Recently, palladium complexes with increased stability are being investigated to circumvent these limitations, and a biphosphinic cyclopalladated complex {Pd_2 _[*S_(-)_*C^2^, N-dmpa]_2 _(μ-dppe)Cl_2_} named C7a efficiently controls the subcutaneous development of B16F10-Nex2 murine melanoma in syngeneic mice. Presently, we investigated the melanoma cell killing mechanism induced by C7a, and extended preclinical studies.

**Methods:**

B16F10-Nex2 cells were treated *in vitro *with C7a in the presence/absence of DTT, and several parameters related to apoptosis induction were evaluated. Preclinical studies were performed, and mice were endovenously inoculated with B16F10-Nex2 cells, intraperitoneally treated with C7a, and lung metastatic nodules were counted. The cytotoxic effects and the respiratory metabolism were also determined in human tumor cell lines treated *in vitro *with C7a.

**Results:**

Cyclopalladated complex interacts with thiol groups on the mitochondrial membrane proteins, causes dissipation of the mitochondrial membrane potential, and induces Bax translocation from the cytosol to mitochondria, colocalizing with a mitochondrial tracker. C7a also induced an increase in cytosolic calcium concentration, mainly from intracellular compartments, and a significant decrease in the ATP levels. Activation of effector caspases, chromatin condensation and DNA degradation, suggested that C7a activates the apoptotic intrinsic pathway in murine melanoma cells. In the preclinical studies, the C7a complex protected against murine metastatic melanoma and induced death in several human tumor cell lineages *in vitro*, including cisplatin-resistant ones. The mitochondria-dependent cell death was also induced by C7a in human tumor cells.

**Conclusions:**

The cyclopalladated C7a complex is an effective chemotherapeutic anticancer compound against primary and metastatic murine and human tumors, including cisplatin-resistant cells, inducing apoptotic cell death via the intrinsic pathway.

## Background

The incidence of malignant melanoma is rising and has not been associated with significantly better therapeutic options. Treatments of choice for the systemic therapy of metastatic lesions have used mono-chemo and immunotherapy, both with low records of clinical response. Even the combination of several chemotherapeutic compounds and the use of biochemotherapy protocols were not able to improve the overall survival of patients [reviewed in [1] and [2]]. In consequence, research directed to the discovery of new antitumor compounds is strongly stimulated.

Most synthetic metal complexes used as antitumor chemotherapeutic compounds are structural analogs of cisplatin. These compounds are frequently associated with severe neuro/nephrotoxicity, myelosuppression side effects, and tumor cell resistance [reviewed in [3]]. Although new analogs designed to circumvent toxic side-effects were not as successful as expected, the research on platinum complexes moved to areas of cancer-specific targeting, drug administration and drug delivery [3]. Recently, new structural types of metallic complexes aiming at increased antitumor efficiency, but also at decreased toxicity in normal cells have been introduced. Simultaneously, new targets in tumor cells that could overcome resistance mechanisms have been discovered. Palladium complexes are among these newly described coordination compounds.

The first palladium complexes had little or no application as antitumor compounds due to poor stability and fast hydrolysis in biological environments. The use of chelating ligands in the synthesis of palladium complexes increased the stability of these compounds [[4], reviewed in [5]].

Recently, palladium complexes have been tested as antimicrobial and antitumor compounds. For example, palladium (II) complexes containing isonicotinamide [6], chiral β-aminoalcohol palladium complexes [7] and palladium(II) oxalate complexes [8] were cytotoxic to human tumor cells *in vitro*, at μM concentrations, but the *in vivo *activity of these compounds in experimental models have not been investigated.

During these studies it was established that the stability of Pd complexes was further improved by the generation of cyclopalladated compounds using cyclometallation reactions [reviewed in [5]]. In addition to the increased stability, cyclopalladated complexes were less toxic, making them promising new antitumor compounds [[9-12], reviewed in [5]].

A biphosphinic palladacycle complex [Pd(C^2^, N-(S_(-)_dmpa)(dppf)] Cl induced apoptotic cell death in human leukemia cells (HL-60 and Jurkat) by rupture of lysosomal membrane and release of cathepsin B in the cytoplasm. The IC_50 _dose after 5 h was 8 μM, and normal human lymphocytes were not sensitive to the complex [13].

Another group of biphosphinic cyclopalladated compounds, obtained from the cyclometallation agents *N*, *N*-dimethyl-1-phenethyl-amine (dmpa), phenyl-2-pyridinyl-acetylene or 1-phenyl-3-*N*, *N*-dimethylamine-propyne and containing the biphosphinic ligand 1, ethanebis (diphenyl-phosphine) (dppe), were synthesized and tested *in vitro *and *in vivo *in syngeneic murine melanoma B16F10-Nex2 cells [14]. In this study with 7 cyclopalladated compounds, 3 complexes inhibited the *in vitro *growth of murine melanoma cells at doses lower than 1.25 μM, and one complex, named C7a, was the most active *in vivo*, delaying subcutaneous tumor growth and increasing animal survival. Cyclopalladated C7a strongly affected the respiratory metabolism of B16F10-Nex2 cells causing a collapse in the mitochondrial proton gradient, suggesting a possible effect on mitochondria, and possibly leading cells to apoptosis, since it was observed DNA degradation [14]. The additive anti-tumor protective effect of the C7a complex in a gene therapy protocol with plasmids encoding IL-12 and an Fc-chimera of the soluble alpha chain of IL-13 receptor was demonstrated by Hebeler-Barbosa *et al*. [15]. The combined therapy rendered a significant reduction in the subcutaneous tumor evolution with 30% tumor-free mice.

It remained to be determined whether human tumor cells or the metastatic experimental model of B16F10-Nex2 cells were sensitive to this new compound and the basis of the antitumor effect, thus validating the C7a complex as a good candidate for clinical trials.

In the present work, we analyzed the mechanism of action of the C7a complex on melanoma cells, determined the *in vivo *effect of cyclopalladated C7a on a preclinical model of metastatic melanoma and the cytotoxicity *in vitro *of the complex on several human tumor cell lines, including some cisplatin-resistant lineages.

## Methods

### Tumor cells and culture conditions

Murine B16F10-Nex2 melanoma cells were cloned at the Experimental Oncology Unit, Federal University of São Paulo, UNIFESP, as described elsewhere [16]. Human melanoma cell line SKmel25 was obtained from the Memorial Sloan Kettering Cancer Center, New York, and all other human cell lines were obtained from the Ludwig Institute for Cancer Research (São Paulo). Tumor cells were cultivated in complete RPMI-1640 medium, pH 7.2, supplemented with 10 mM *N*-2-hydroxyethylpiperazine-*N*'-2-ethanesulphonic acid (HEPES), 24 mM sodium bicarbonate, 40 mg/ml gentamycin, 100 U/mL penicillin, 100 μg/ml streptomycin and 10% fetal calf serum (FCS), all from Invitrogen (CA, USA). Cells were maintained in culture flasks at 37°C in humidified atmosphere with 5% CO_2_, and were collected using PBS-EDTA (1 mM) solution.

### Animals

C57Bl/6 male mice (8 weeks old) were purchased from CEDEME (Centro de Desenvolvimento de Modelos Experimentais, UNIFESP), and maintained in sterilized environment, with food and water *ad libitum*, in 12 h cycles of light/dark. All animal experiments were approved by the Animal Experimentation Ethics Committee of UNIFESP, under protocol No. 1507/09.

### Cyclopalladated compound

**Figure 1:**
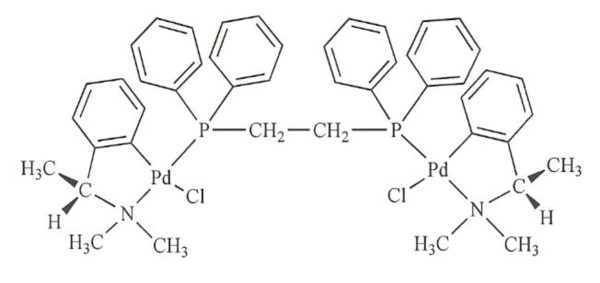
**Schematic representation of the cyclopalladated C7a complex, [Pd_2_(S*_(-)_*C^2^, N-dmpa)_2_(μ-dppe)Cl_2_]**.

The cyclopalladated complex C7a was synthesized from *N*, *N*-dimethyl-1-phenethylamide (dmpa), complexed to 1, 2 ethanebis (diphenylphosphine, dppe) ligant, as previously described in Rodrigues *et al*. [14] and its chemical formula is shown in Figure 1. The compound is diluted to a final concentration of 10 mM in DMSO (cell culture tested, Sigma Aldrich), and for *in vivo *and *in vitro *assays diluted to the final concentration in complete RPMI-1640 medium.

### In vitro *Cell Viability Assay*

Tumor cells were seeded at 10^4 ^cells/well into 96 well-plates (Corning Costar Co, NY, USA), and 12 h later, they were incubated with serially diluted C7a to a final volume of 200 μL in complete RPMI medium. After 24 h incubation with C7a, the cytotoxic activity was determined by measuring cell viability by two different methods, Trypan Blue exclusion and the Cell Proliferation kit (MTT, Roche Diagnostics Comp., Indianopolis, IN), following the manufacturer's instructions. A 50% inhibition of cell growth was taken as a comparative index of cytotoxicity (IC_50_). To verify the inhibitory effect of DTT on C7a cytotoxic effect, cells were pre-incubated with 2 mM dithiothreitol (DTT, Sigma Aldrich, MO) for 10 minutes, and then with a high dose of C7a (10 μM) for 1 or 2 h. Alternatively, cells were incubated for 2 h with DTT, carefully washed with serum-free medium and then incubated with 1 μM C7a for 1 and 2 h. Viable cells were counted by Trypan Blue exclusion, and each IC_50 _value was calculated using at least 3 separate experiments.

### Isolation of rat liver mitochondria (RLM)

Mitochondria were isolated by conventional differential centrifugation [17] from the liver of adult rats. Male Wistar rats weighing approximately 180 g were sacrificed by cervical dislocation and the liver was immediately removed and homogenized in 250 mM sucrose, 1 mM EGTA, and 10 mM HEPES-KOH buffer (pH 7.2) in a Potter-Elvehjem homogenizer. Homogenates were centrifuged at 770 *g *for 5 min and the resulting supernatant was further centrifuged at 9800 *g *for 10 min. Pellets were suspended in the same medium containing 0.3 mM EGTA and centrifuged at 4500 *g *for 15 min. The final pellet was resuspended in 250 mM sucrose and 10 mM HEPES-KOH buffer (pH 7.2) to a final protein concentration of 80-100 mg/ml. All studies with mitochondria were performed within 3 h and mitochondrial protein content was determined by the Biuret reaction [18].

### Mitochondrial swelling

Rat liver mitochondria (0.25 mg protein/ml) were suspended in a medium containing 125 mM sucrose, 65 mM KCl, 10 mM HEPES-KOH, pH 7.4, at 30°C plus 5 mM potassium succinate, 2.5 μM rotenone and 10 μM CaCl_2_. Complex 7a was added 10 seconds after the start of data recording. The mitochondrial swelling was estimated from the decrease in the relative absorbance at 540 nm in a Hitachi U-2000 Spectrophotometer (Tokyo, Japan).

### Measurement of mitochondrial membrane potential (ΔΨ) in isolated mitochondria

Mitochondrial ΔΨ was estimated under the same experimental conditions of the swelling assay. Changes of 0.4 μM rhodamine 123 fluorescence were recorded on a Hitachi F-2500 Spectrofluorometer (Tokyo, Japan) operating at 505/525 nm with a slit width of 5/5 nm, excitation/emission, respectively. The results are expressed as percentage of dissipation in relation to uncoupled mitochondria (carbonyl cyanide trifluoro-methoxyphenylhydrazone, FCCP, Sigma Chemical, MO, 1.0 μM).

### Measurement of extracellular acidification rate

HCT-8, SiHa and SKmel25 human tumor cells (3 × 10^5^) were seeded on 3 μm pore transwells (Corning Costar), and grown in complete culture medium containing 10% FCS in 12-well culture plates for 12 h before the experiment. The extracellular acidification rate of C7a-treated and untreated cells was determined using a Cytosensor Microphysiometer (Molecular Devices, Gräfelfing, Germany). Capsules containing the adherent cells were transferred to sensor chambers and kept in a low buffered RPMI containing 1% BSA at 37°C for 20 min, until extracellular acidification rate stabilization, producing a basal line. The perfusion medium was then pumped through each sensor chamber at 50 μl/min, and pumping cycles consisted of a flow period of 90 sec, followed by a flow-off period of 30 sec. During these periods, protons released from the cells accumulated in the sensor chamber, and the slope of the H^+ ^profile was quantified every 2 minutes. Cells were perfused with low-buffered medium containing 1% BSA (control), 10 μM C7a or 200 μM Cisplatin, both compounds diluted in the same medium. Compounds were maintained until the end of the experiment.

### ΔΨm (mitochondrial transmembrane potential) measurement in intact tumor cells

Mitochondrial membrane potential measurements were carried out as described previously [19]. Briefly, 5 × 10^4 ^B16F10-Nex2 cells were seeded on 25 mm^2 ^poly-lysine (1 mg/ml) pre-treated coverslips. After cell attachment, coverslips were placed in Leiden coverslip chambers, and cells were incubated with 50 nM of TMRE (tetramethylrhodamine ethyl ester, Molecular Probes, OR, USA) for 15 minutes at room temperature. The apparatus was transferred to a thermostatically regulated microscope chamber (37°C, Harvard Instruments, MA, USA) and 1 μM C7a, or alternatively 10 μM C7a in the presence of 2 mM DTT, was added. TMRE fluorescence (548 nm excitation and 585 nm emission) was acquired immediately after C7a addition at 1 frame/6 seconds using a TE300 Nikon inverted microscope (Nikon Osaka, Japan) and a 16 bit cooled CCD camera CoolSnap (Roper Sci, Princeton Instruments, USA) controlled by imaging software (BioIP, Wilmington, DA). Because of the high resolution, individual mitochondria were localized, especially at the borders of the cells, and the regions of interest (ROI) were drawn surrounding each mitochondrion. For calibration, at the end of each experiment (after 90-100 images captured) 5 μM FCCP (Sigma Chemical, MO, USA), a protonophore uncoupler that collapses ΔΨm, was added. Fluorescence intensity was measured in arbitrary units. At least 100 mitochondria/coverslip and nine coverslips/treatment were analyzed. In addition, cells showing depolarization (decrease), hyperpolarization (increase) or no change in the mitochondrial fluorescence were visually counted. The assay was done in triplicate, and at least 200 cells were counted in each slide.

### Bax translocation

B16F10-Nex2 cells (5 × 10^4^) were seeded on 25 mm^2 ^poly-lysine (1 mg/ml) pre-treated coverslips. After attachment, cells were transfected with a GFP-Bax plasmid as described previously [20]. Briefly, 0.5 μg of GFP-Bax plasmid and 4 μl of LipofectAmine (Life Technologies, Gaithersburg, MD, USA) were used per coverslip. Cells were incubated for 5 h in the transfection mixture at 37°C, placed in Leiden coverslip chambers and adapted to the microscope, where the temperature of the specimen was maintained at 35-37°C. The complex C7a (1 μM) was then added and GFP-Bax translocation from cytosol to intracellular compartments was observed using an inverted confocal microscope LSM510 (Carl Zeiss, Heidelberg, Germany) equipped with ArKr 488/568, HeNe 543 lasers and 40 × Apochromat objective. Alternatively, intracellular calcium was chelated with 20 μM 1,2-Bis(2-aminophenoxy)ethane-*N,N,N',N'*-tetraacetic acid tetrakis (acetoxymethyl ester) (BAPTA-AM, Sigma Chemical, MO, USA) for 20 minutes before C7a addition. For mitochondrial colocalization of Bax, transfected cells were loaded with 20 nM tetramethylrhodamine ethyl ester perchlorate (TMRE) for 10 minutes previously to the addition of C7a complex. Time course analyses of treated cells were carried out at 30 min intervals to monitor changes in GFP-Bax and TMRE localization

### Calcium measurements assay

B16F10-Nex2 cells (5 × 10^4^) were seeded on 25 mm^2 ^poly-lysine (1 mg/ml) pre-treated coverslips. After cell attachment, coverslips were incubated with 2 μM Fura-2-AM (Molecular Probes, OR, USA) plus 20% Pluronic F127 (Sigma Chemical, MO, USA) for 30 minutes at room temperature. The coverslips were then washed and placed in Leiden coverslip chambers, adapted to the microscope, where the temperature of the specimen was maintained in 35-37°C. Cytosolic concentration of calcium on untreated cells was normalized to the zero value of Fluorescence Ratio 340/380 nm. Complex C7a (1 μM) was then added (Image number Zero) and images were collected at 3 sec intervals by using a TE300 Nikon inverted microscope (Nikon Osaka, Japan) coupled to 16 bit cooled CCD camera CoolSnap (Roper Sci, Princeton Instruments, USA) controlled by imaging software (BioIP, Wilmington, DA). Fura-2, a ratiometric calcium dye, was excited at 340 and 380 nm with emission acquired at 505 nm. Readings at 340 and 380 nm were used to calculate fluorescence ratios, which represented the variations in cytosolic calcium under these circumstances. Single cells were then analyzed using the ROI tool, fluorescence intensities obtained were normalized and plotted using the BioIP software and Kaleida Graph Synergy software. The effect of C7a was evaluated in the presence or absence of external calcium, and the maximum effects evoked by C7a under these conditions were plotted in a histogram. Seven cells/coverslip from at least nine different experiments were analyzed.

### Quantification of cytosolic ATP

Cytosolic ATP concentration was measured using a bioluminescence assay kit (*Adenosine 5'-triphosphate bioluminescent somatic cell assay kit*, Sigma-Aldrich). Briefly, 2 × 10^4 ^plated cells were treated with 1 μM C7a (100 μl) for 15, 30, 45 or 60 minutes. Cells (or medium, as control) were lysed in 100 μl of ATP-releasing reagent and 50 μl of this suspension was added to 50 μl of ATP assay mix solution into each well of white 96-well plates. Light emission was measured at 570 nm in a SpectraMaxL luminometer (Molecular Devices, CA, USA). A standard curve obtained with diluted ATP solutions was used to calculate ATP concentrations in samples.

### Identification of activated caspases

The *ApoTarget*™ *Caspase Colorimetric Protease Assay kit *(Invitrogen, CA, USA) was used for measurement of caspase-2, caspase-3, caspase-6, caspase-8 and caspase-9 activities in cell lysates. Briefly, B16F10-Nex2 cells (5 × 10^6^) were treated *in vitro *with 1 μM C7a for 5 minutes. As a positive control, B16F10-Nex2 cells were exposed to ultraviolet (UV) light for 7 minutes. Cell lysates were obtained following manufacturer's instructions and caspase activity was measured at 400-405 nm as free p-nitroaniline released from p-nitroaniline-labeled specific substrates. Alternatively, caspase-3 activity was measured by flow citometry. B16F10-Nex2 cells (1 × 10^6^) were treated for 12 h with 1 μM C7a or 10 μM Actinomycin D, as a positive control. The cells were collected and incubated with Anti-ACTIVE^® ^Caspase-3 policlonal antibody (Promega, WI, USA) diluted 1:1000 in PBS/BSA 1% for 1 h on ice and maintained in the dark. Samples were evaluated in a FACScalibur Flow Cytometer (BD Biosciences, CA, USA), using CellQuest^® ^software.

### Analysis of nuclear alterations

B16F10-Nex2 cells (5 × 10^4^) were grown for 12 h on sterile 25 mm^2 ^coverslips in 6-well tissue culture plates. Cells were treated with 1 μM C7a and coverslips collected at different times were washed with PBS and stained with Hoechst 33342 (Sigma-Aldrich, MO, USA) for 15 minutes. Coverslips were inverted on slides and analyzed in an Olympus BX61 microscope (magnification 400 ×) at 360 nm. The images were acquired using Cell^M Software. Nuclear alterations in at least 200 cells for each time point were visually observed and counted, and the frequency of cells showing alterations was calculated. The assay was repeated three times.

### Morphological analyses

For analysis of morphological changes in tumor cells by light microscopy, B16F10-Nex2 cells (1 × 10^4^) were grown for 12 h on sterile 13 mm^2 ^coverslips inserted into 12-well tissue culture plates (Corning Costar). Cells were treated with 1 μM C7a and coverslips collected at different times were washed in PBS, inverted on glass slides and analyzed in an Olympus BX61 microscope (magnification 1000 ×). The images were acquired using Cell^M Software. For transmission electron microscopy (TEM) analysis, B16F10-Nex2 cells (5 × 10^4^) were grown for 12 h on sterile 13 mm^2 ^coverslips in 12-well tissue culture plates. Cells were treated with 1 μM C7a for 15 min and fixed in glutaraldehyde 2.5% in sodium cacodylate buffer 0.1 M, pH 7.4. The post-fixation was performed using 1% osmium tetroxide, 0.08% potassium ferricyanide, 5 mM calcium chloride in the same buffer for 60 minutes in the dark. Dehydration was made in series of acetone and infiltration in polybed epoxy resin (Polysciences, PA, USA). Ultrathin sections obtained by ultramicrotomy were collected in grids (300 mesh) and contrasted in uranyl acetate and lead citrate. The ultrastructural analysis was done in a transmission electron microscope Zeiss EM-900.

### Treatment of Experimental Tumor Metastasis

C57Bl/6 mice were injected intravenously into the tail vein with 5 × 10^5 ^B16F10-Nex2 viable cells in 100 μL of RPMI medium. Intraperitoneal injections of C7a (200 ng·kg^-1^) or PBS (control group) started 24 h after tumor inoculation, and treatment was repeated 3 times a week for 13 days. Animals were killed by cervical dislocation on day 15^th^, lungs were collected and pulmonary nodules were counted using an inverted microscope.

### Statistical analysis

Statistical analysis was performed using Student's t Test from Microsoft Excel (Microsoft Office Software). Values (*p*) equal to or less than 0.05 were considered significant.

## Results

### C7a complex enters passively tumor cells in vitro and affects cell morphology

Murine melanoma B16F10-Nex2 cells were incubated with Complex C7a at 4°C or 37°C, and no difference in the cytotoxicity of C7a at both temperatures was observed (Additional file 1, Figure S1). This result suggests that Complex C7a enters passively the tumor cells, independently of a specific receptor.

The cell morphology of B16F10-Nex2 cells treated with C7a was examined by light microscopy, and changes occurred very early after treatment. After 5 minutes incubation with C7a, B16F10-Nex2 cells had some detached cells, and an increased cytoplasmic granulation associated with nuclear condensation. Alterations were time-dependent (Additional file 2, Figure S2).

### Complex 7a has a direct effect on isolated mitochondria

**Figure 2:**
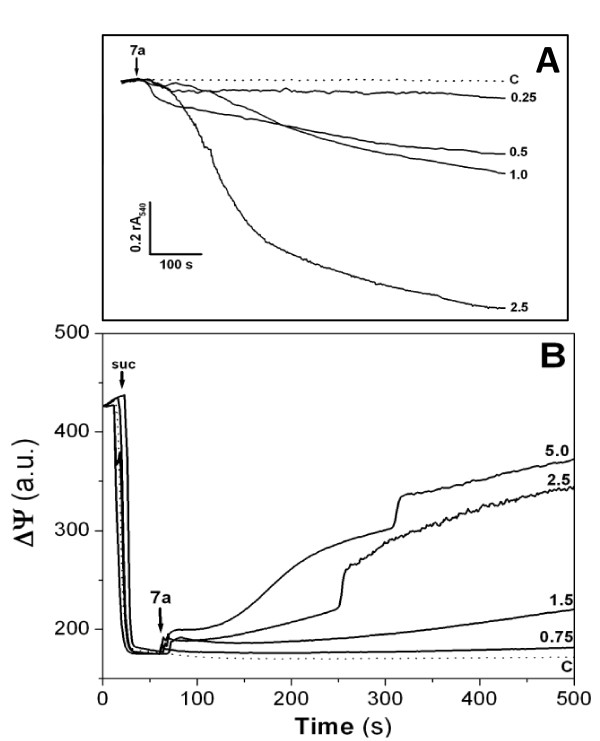
**Complex 7a-induced mitochondrial permeabilization results in swelling and electrochemical gradient dissipation on isolated rat liver mitochondria**. **(A) **Mitochondrial swelling was estimated by decrease of the relative absorbance recorded at 540 nm (rA540); **(B) **ΔΨ was measured by using 0.4 μM rhodamine 123 and mitochondria were energized by the addition of 5 mM succinate (suc). The arrows indicate the addition of Complex 7a, the numbers are the cyclopalladated compound concentrations at μM, and the traces are representative of at least three experiments with different mitochondrial preparations. **C **represents untreated mitochondria (in dotted lines).

The direct effects of Complex 7a on isolated rat liver mitochondria were investigated. As observed in the Figure 2A, the addition of the palladacycle on mitochondrial suspension energized with potassium succinate resulted in a concentration-dependent swelling. This effect started at 250 nM and reached the maximal effect at 2.5 μM concentration. Such swelling indicates the occurrence of the inner mitochondrial membrane permeabilization associated with the disruption of ionic homeostasis in mitochondria. In this way, it was also observed the dissipation of the mitochondrial transmembrane potential induced by the addition of Complex 7a to the mitochondrial suspension due to the permeabilization promoted by the cyclopalladated compound (Figure 2B).

### Cyclopalladated C7a induces a collapse of the mitochondrial membrane potential (ΔΨm) by interacting with protein-thiol groups in intact tumor cells

**Figure 3:**
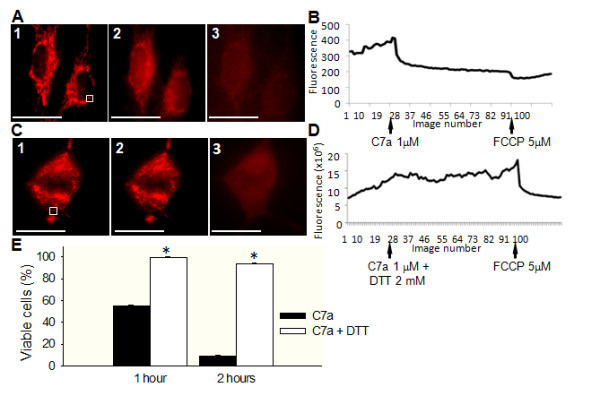
**Effect of C7a on mitochondrial membrane potential in intact cells, and inhibitory effect of DTT on C7a**. (A1 and C1) Representative image of *Δ*Ψm in B16F10-Nex2 cells in resting conditions after incubation with TMRE. White squares represent one ROI, as described in Material and Methods; **(A2) **Representative image of TMRE-incubated cells immediately after C7a addition; **(A3) **Representative image of TMRE/C7a-treated cells immediately after FCCP addition. Scale bars, 20 μm. Images show one of 30 similar images that were acquired at 1 frame/6 seconds, 400 × magnification. **(B and D) **Representative traces of TMRE fluorescence over time in B16F10-Nex2 cells, arrows show the moment C7a or FCCP were added to the cells. **(C2) **Representative image of TMRE-incubated tumor cells, pretreated with DTT and immediately after C7a addition. **(C3) **Representative image of TMRE/C7a/DTT-treated tumor cells immediately after FCCP addition. Scale bars, 20 μm. Images were acquired as described in (A). **(E) **Cytotoxic effect of C7a in the presence or absence of DTT. The percentage of viable cells related to untreated control is represented. *p < 0.05, compared to untreated cells at the same time. Each experiment was performed at least 3 times.

B16F10-Nex2 cells were incubated with TMRE, a fluorescent probe used to measure ΔΨm variations. In resting conditions, mitochondria appear as elongated structures regularly distributed in the cell cytoplasm. C7a-treated cells showed a rapid and strong decrease in fluorescence intensity that was sustained until FCCP was added. FCCP was used to promote the collapse of ΔΨm (Figure 3A). The histogram in Figure 3B is representative of the ΔΨm variations throughout the assay of one single organelle from treated cells. As shown in Table 1, the majority of mitochondria analyzed in C7a-treated cells (88%) showed a decrease in TMRE fluorescence (depolarization), while the remaining showed an increase (hyperpolarization) or no effect on TMRE fluorescence after treatment with the cyclopalladated compound.

**Table 1:** Frequency of B16F10-Nex2 cells showing alterations in mitochondrial membrane potential after C7a or C7a + DTT treatment.

	*C7a (%)*	*C7a+ DTT (%)*
***Depolarization***	88.0 ± 1.5	6.0 ± 0.6
***Hyperpolarization***	11.0 ± 2.5	9.0 ± 1.6
***No change***	1.0 ± 1.5	85.0 ± 2.0

Recently, Santana *et al*. [21] demonstrated that an enantiomer of cyclopalladated C7a {Pd_2 _[*R_(+)_*C^2^, N-dmpa]_2 _(μ-dppe)Cl_2_} interacts with thiol groups of the membrane proteins from rat liver isolated mitochondria, promoting specific protein thiol cross-linkage, inducing mitochondrial permeabilization and cytochrome *c *release. This effect was abolished by preincubation of isolated mitochondria with DTT, a thiol reducing agent. To analyze whether C7a could induce the same effect on intact tumor cells, B16F10-Nex2 cells were incubated *in vitro *with C7a in the presence of DTT. The cytotoxic effect of C7a on B16F10-Nex2 cells was significantly inhibited by DTT, with more than 90% of treated cells remaining viable after 1 h and 2 h incubation (Figure 3E). Corroborating this result, the rapid ΔΨm dissipation induced by C7a was completely inhibited by DTT (Figure 3C, D and Table 1). To discard a possible quenching reaction between C7a complex and DTT prior to cell interaction, since DTT has free SH groups in its structure, B16F10-Nex2 cells were pretreated with DTT for 2 h, carefully washed with serum-free medium and then incubated with C7a. In this experimental setting, DTT inhibited the cytotoxic effect of C7a as observed previously (data not shown).

### Bax-translocation to mitochondria induced by C7a

Bax is a pro-apoptotic member of Bcl-2 protein family located predominantly in the cytosolic compartment, and in monomeric form in healthy cells. After an apoptotic stimulus, a significant fraction of Bax protein translocates to the outer mitochondrial membrane [22] and after insertion into mitochondria, Bax may form large oligomers at the membrane.

**Figure 4:**
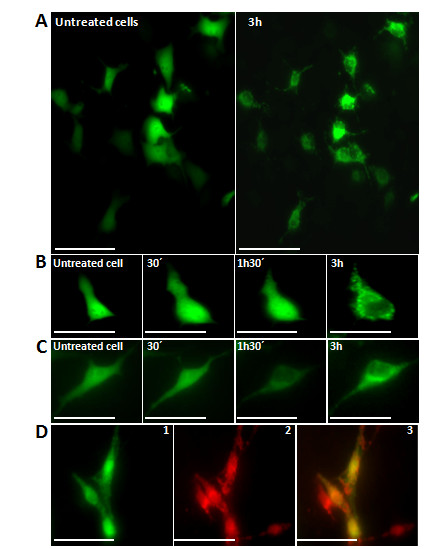
**Bax translocation to mitochondria in GFP-Bax-transfected B16F10-Nex2 cells after treatment with C7a is dependent on Ca^2+^**. **(A) **Representative images of untreated cells and after 3 h treatment; **(B) **Representative images after selected time points of an individual tumor cell; **(C) **The same assay performed in (B) with transfected cells preincubated with 20 μM of BAPTA before C7a addition; **(D) **Representative images show Bax-transfected C7a-treated cells after 2 h (1), co-stained with TMRE (2) and overlayed image (3). Scale bars, 20 μm in (A), 40 μm in (B) an (C) and 30 μm in (D). Images are representative of at least three individual experiments.

To verify if C7a was capable of activating Bax inducing protein translocation, B16F10-Nex2 cells were transfected with a recombinant GFP-Bax plasmid and treated with the complex. Figure 4A, B shows that C7a evoked a punctuated pattern characterized by intense dotted profile indicating that the compound induced Bax-translocation to mitochondria, as observed by the colocalization with TMRE (Figure 4D). Untreated cells showed a diffuse pattern of GFP fluorescence characteristic of soluble Bax in the cytosol.

### Calcium contibutes to cell death caused by C7a

**Figure 5:**
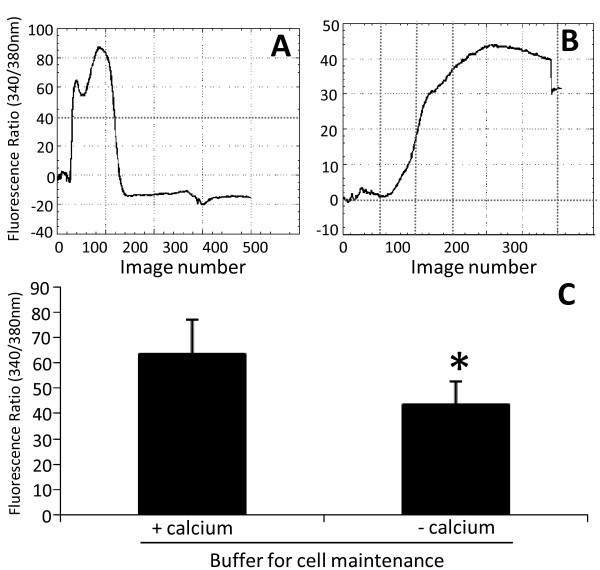
**Increase in cytosolic calcium induced by C7a**. Calcium concentration was evaluated in B16F10-Nex2 cells incubated with regular or calcium-free buffer. **(A) **and **(B) **Representative fluorescence ratio (340/380) traces of single cells are shown after C7a (1 μM) addition at Image Zero in the presence (A) or absence of calcium in the buffer (B). **(C) **Histogram shows the media ± standard deviation of the fluorescence ratio at 340/380 in all cells analyzed. Normalized fluorescence value was determined before C7a addition and data were expressed as percentage in relation to the baseline. *, p < 0.05. The results are representative of 3 independent experiments.

Calcium transients are important events for cell physiology and the regulation of this signaling process depends on a complex machinery, which maintains cytosolic calcium at nanomolar levels in resting conditions [23]. Increase in intracellular calcium concentrations may trigger apoptotic pathways or even amplify other apoptotic stimuli. C7a treatment of B16F10-Nex2 cells induced a significant increase in cytosolic calcium (Figure 5A), which may be released mainly from intracellular organelles, such as mitochondria and endoplasmic reticulum, since the effect was present even when cells were incubated in a calcium-free buffer (Figure 5B). In Figure 5C, the histogram shows the maximum increase in Fura-2 ratio induced by C7a in the presence or absence of calcium. In the presence of a regular buffer (with calcium), the cytosolic calcium response was even greater indicating that the extracellular calcium may also participate in C7a-induced calcium increase. Fluorescence intensities were not changed and remained stable in the absence of C7a (data not shown).

To corroborate the participation of Ca^+2 ^on the mitochondrial effect of C7a complex, B16F10-Nex2 murine melanoma cells were pre-incubated for 20 minutes with BAPTA-AM, an intracellular calcium chelator, before C7a addition and in a Ca^+2^-containing medium. It was observed that Bax was unable to translocate to mitochondria in this condition, as evidenced by the maintenance of the original diffuse pattern (Figure 4C).

### C7a treatment of intact tumor cells causes mitochondrial swelling and decreased ATP production

Our results suggest that the mitochondrial membrane of intact tumor cells was strongly affected by C7a treatment, by opening the mitochondrial permeability transition pore complex (PTPC) in association with Bax polymerization at the membrane. The pore-forming effect associated with the rapid collapse of the ΔΨm caused by cyclopalladated C7a, may induce several other modifications in mitochondria, such as uncoupling of ATP synthesis from oxidative phosphorylation and mitochondrial swelling [24].

**Figure 6:**
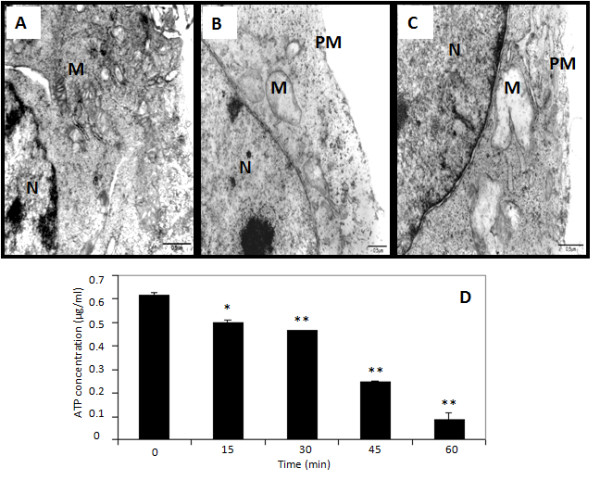
**Transmission electron microscopy and intracellular ATP concentration after C7a treatment**. **(A) **Untreated control cells; **(B and C) **C7a treated cells. **N**, nucleus; **M**, mitochondria; **PM**, plasma membrane. Scale bars, 0.5 μm; **(D) **B16F10-Nex2 cells were treated with C7a and the ATP levels were evaluated at different time points, as described in Materials and Methods. *, *p < 0.05 *and **, *p < 0.01*, compared to cells at time zero. Results are representative of 3 independent experiments.

Swelling of mitochondria could be observed in transmission microscopy images of treated cells. Untreated B16F10-Nex2 cells showed intact mitochondria with elongated shape and preserved lamellar cristae (Figure 6A). In contrast, C7a-treated cells showed swollen mitochondria with irregular shape, undefined cristae and ruptured membranes (Figures 6B, C).

Mitochondria swelling and collapse of ΔΨm results in the arrest of ATP synthesis by these organelles, and in fact, treatment with C7a did affect ATP synthesis in B16F10-Nex2 cells. More than 30 minutes incubation with the compound was, however, necessary to reduce 50% ATP concentration. After 1 h, 15% of the initial concentration of ATP was still detected (Figure 6D).

### C7a treatment releases caspase-activators and endonucleases from mitochondria in intact tumor cells

Alterations in mitochondrial permeability may release numerous proteins that are normally confined within the mitochondrial intermembrane space where they exert vital functions. Among them are caspase activators and endonucleases [25].

**Figure 7:**
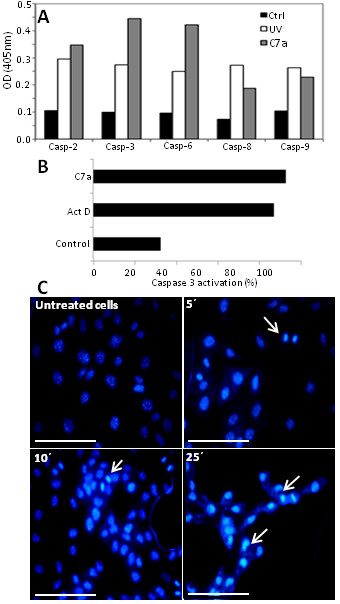
**Caspase activation and nuclear alterations after treatment with C7a**. **(A) **Activation of caspases after C7a treatment measured by a colorimetric assay. **Ctrl**, untreated tumor cells; **UV**, positive control, cells submitted to UV irradiation. **(B) **Percentage of activated caspase-3-positive cells in flow citometry after **Actinomycin D **or **C7a **treatment. **Control**, untreated tumor cells incubated with anti-active caspase 3 antibody. **(C) **Representative images of C7a-treated B16F10-Nex2 cells after nuclear staining with Hoescht 33342. Arrows represent nuclear alterations. Magnification, 400 ×, and scale bars, 20 μm. Results are representative of at least 2 independent experiments.

In B16F10-Nex2 cells all caspases tested were activated by C7a, including the effector caspases 3 and 6, responsible for the final events of apoptosis (Figure 7A, B). As positive control, exposure to ultraviolet (UV) radiation also induced activation of caspases. Interestingly, the levels of effector caspases 2, 3 and 6 activation were higher in cyclopalladated C7a-treated cells compared to UV treatment (Figure 7A).

C7a treatment also caused release of endonucleases from mitochondria, with the expected nuclear alterations and DNA degradation. Significant nuclear alterations were observed in C7a-treated cells, as observed after Hoechst 33342 staining. While no significant chromatin condensation was observed in untreated cells, picnotic nuclei were observed in 85% cells after 25 minutes incubation with C7a, and this effect was time-dependent (Figure 7Cand Table 2).

**Table 2:** Frequency of cells showing nuclear alterations after C7a treatment.

*Time (min)*	*Cells with nuclear alterations (%)*
0	6.5 ± 0.4
5	12.5 ± 1.1
10	41 ± 1.5
25	85 ± 0.9

### Cyclopalladated C7a was effective in a pre-clinical model of metastatic melanoma

**Figure 8:**
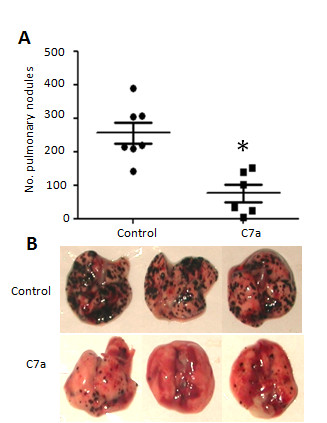
**Antimetastatic activity of C7a**. C57Bl/6 mice injected endovenously with B16F10-Nex2 cells were treated i.p. with C7a. **(A) **Number of pulmonary nodules 15 days after tumor inoculation, each symbol represents one individual animal. Horizontal lines represent the average and standard deviation; (**B) **Representative images of lungs from treated (**C7a**) and untreated (**Control**) animals. * *p < 0.05*. Results are representative of 3 independent experiments.

To analyze the effect of C7a on metastatic melanoma growth, C57Bl/6 mice were injected endovenously with tumor cells and intraperitoneally treated with C7a for 13 days. The number of lung nodules was counted after 15 days. The treatment protocol significantly reduced the number of lung nodules (Figure 8).

### Cyclopalladated C7a was cytotoxic in vitro against human tumor cells and affected the respiratory metabolism

The cytotoxic effect of C7a was tested *in vitro *against several human tumor cell lines, and viable cells were determined by two different methods (Table 3). The effect was dose-dependent (not shown), and the IC_50 _values for the majority of human tumor cell lines tested were below 400 nM. Me91 and SKMel25 were the most sensitive cell lines, both isolated from human melanomas. The IC_50 _for B16F10-Nex2 murine cell line was 420 nM, superior to the values obtained for sensitive human tumor cell lines. Human leukemia (HL-60) and glioblastoma (U-97) cell lines showed the highest IC_50 _values, 1.0 and 3.5 μM, respectively. Cisplatin, a standard chemotherapeutic compound, was cytotoxic in some human cell lines, with IC_50 _values ranging from 66 to 176 μM. Some human cell lines tested were significantly resistant to cisplatin, with IC_50 _values superior to 200 μM. Remarkably, C7a compound was cytotoxic in these cisplatin-resistant cells (LS-180, HCT-8, ZR753A, SiHa and SKMel25).

**Table 3:** Comparison of IC_50 _values obtained for C7a and cisplatin

	*IC_50_* *Complex 7a (μM)*	*IC_50_* *Cisplatin (μM)*
***Murine Melanoma***		
B16F10-Nex2 ***Human tumor lineages***	0.42 ± 0.06	176 ± 15
***Colon Cancer***		
LS-180	0.26 ± 0.07	>200
HCT-8	0.34 ± 0.05	>200
***Breast Cancer***		
SKBr-3	0.26 ± 0.04	66 ± 7
ZR753A	0.34 ± 0.02	>200
***Uterine Cervix Cancer***		
HeLa	0.30 ± 0.03	68 ± 4
CasKi	0.24 ± 0.01	140 ± 8
SiHa	0.19 ± 0.02	>200
***Melanoma***		
Me91	0.14 ± 0.03	156 ± 8
SK-Mel 25	0.15 ± 0.01	>200
***Leukemia***		
HL-60	1.0 ± 0.1	70 ± 5
***Glioblastoma***		
U-87	3.5 ± 0.2	150 ± 20

**Figure 9:**
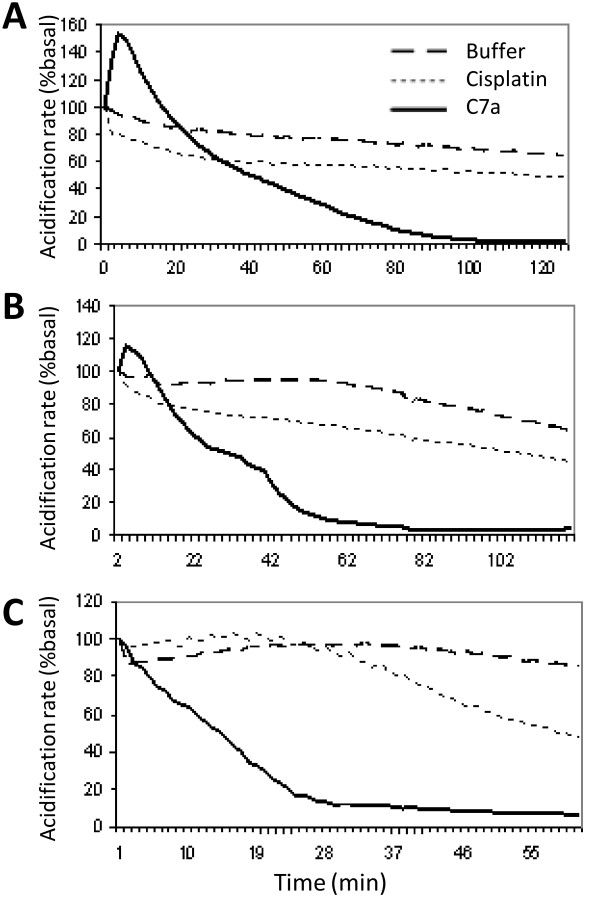
**Acidification response of human cancer cells to C7a**. Cells were placed on the Cytosensor System and allowed to equilibrate in low buffered RPMI containing 1% BSA to normalize the acidification rate in 100% (**Buffer**). **C7a **and **Cisplatin **were diluted in the same medium, added at zero time and maintained during the whole assay. Cells were monitored every 2 min. **(A) **HCT-8 cells; **(B) **SiHa cells; **(C) **SKmel25 cells. Results are representative of 2 independent experiments.

The C7a complex induced an abrupt inhibition of the respiratory metabolism in B16F10-Nex2 cells [14], and this result suggested that mitochondria were involved in the cell death process, which was confirmed by the experiments described here. The same parameter was therefore analyzed in some C7a-sensitive human tumor cell lines, by measuring the effect of the cyclopalladated compound on the extracellular acidification rates using the Cytosensor microphysiometer. While untreated cells maintained baseline values of extracellular H^+ ^throughout the experiment, C7a-treated human tumor cells showed an abrupt decay of extracellular acidification values, similarly to those observed in murine melanoma cells (Figure 9). The respiratory metabolism of human tumor cells was slightly affected by cisplatin.

## Discussion

Elucidating the mechanism of action of a cytotoxic compound can be a challenging task. For example, 40 years have passed since the discovery of the anticancer activity of cisplatin, and the mechanism of action of this compound is still unclear. It is now believed that cisplatin enters the cell by passive diffusion and by copper transporter hCtr1 (human Cu transporter 1) pathways [26]. Once inside the cytoplasm, the low chloride concentration causes cisplatin to undergo aquation and hydrolysis reactions generating various positively charged platinum species. The aquated platinum complexes are believed to bind to nuclear DNA or to other weak nucleophiles such as carbonate and phosphates, but it has been demonstrated that only a small fraction of intracellular cisplatin binds to nuclear DNA triggering the events that will lead to cell death. On the other hand, cisplatin and the aquated complexes react readily with methionine, metallothioneins and glutathione (GSH), and this is believed to be the cause of the high rate of resistance induced by this compound [reviewed in [27]]. The mechanism or mechanisms by which nuclear cisplatin/DNA adducts generate the cytoplasmic events leading to apoptosis have not been defined yet [28]. However, recent reports demonstrate that cisplatin binding to nuclear DNA is not necessary for induction of apoptosis, and that mitochondria may be the main target on tumor cells, although the precise target in these organelles is not known [reviewed in [28]].

Mitochondria in tumor cells are structurally and functionally different from the organelles in non-transformed cells, and recently they are emerging as a selective target for tumor chemotherapy [29].

Santana *et al*. [21] showed that a cyclopalladated compound targets mitochondria. Four cyclopalladated compounds derived from dmpa complexed to dppe were tested against hepatoma cells and shown to be cytotoxic *in vitro*. The most effective compound was the *R(+) *enantiomer of C7a complex, {Pd_2 _[*R_(+)_*C^2^, N-dmpa]_2 _(μ-dppe)Cl_2_}. This *R(+) *enantiomer of C7a was further tested in isolated rat liver mitochondria, causing mitochondrial permeabilization, as indicated by Ca^+2^- and ROS-independent mitochondrial swelling, and release of cytochrome *c*. Associated with the mitochondrial swelling, dissipation of mitochondrial transmembrane potential, uncoupling of the oxidative phosphorylation, and mitochondrial calcium release were observed. These effects were completely inhibited by dithiothreitol (DTT). Importantly, it was observed a decrease in the content of reduced thiol groups in the mitochondrial membrane proteins of treated cells, as well as the presence of membrane protein aggregates without lipid and GSH oxidation. This effect was related to the formation of disulfide bonds between critical thiol groups in mitochondrial membrane proteins, suggesting that cyclopalladates could act as thiol reagents, catalysing specific protein thiol cross-linkage and initiating mitochondrial effects. Interestingly, cyclopalladated-induced mitochondrial permeabilization depended on the entire structure of the compound and not on the palladium atom alone, since PdCl_2 _was not able to promote permeabilization. It is noteworthy that the *R(+) *enantiomer of C7a, although cytotoxic *in vitro*, was not effective *in vivo *against murine melanoma B16F10-Nex2, possibly because of the presence of a *S(-) *anomeric carbon on C7a that stabilizes the molecule by an intramolecular hydrogen bridge formation [14,21]. To compare with the *R(+) *enantiomer, the mechanism of action of the cyclopalladate complex C7a, that was otherwise effective *in vivo*, was investigated.

As observed for cisplatin [30,31], we found little difference in the cytotoxicity of C7a in murine melanoma B16F10-Nex2 cells at 4°C or 37°C, suggesting a passive entry of the compound in tumor cells.

Similarly to the *R(+) *enantiomer, we show here that the C7a complex induced swelling of rat isolated mitochondria and dissipation of the mitochondrial transmembrane potential, associated with inner mitochondrial membrane permeabilization.

The effect of C7a on intact B16F10-Nex2 cells was completely inhibited by DTT, as observed for the *R(+) *enantiomer on isolated mitochondria. This result suggests that C7a may also induce mitochondrial permeabilization by catalyzing the cross-linkage of vicinal thiol groups present in the mitochondrial membrane proteins with the formation of disulfide bonds.

The cross-linkage of thiol groups in mitochondrial membrane proteins was associated to the mitochondrial permeability transition pore complex (PTPC) formation and opening [32-34]. Many modulators of PTPC opening are known, however, the biochemical nature of the proteins assembled to form the PTPC is not completely elucidated. A proposed model suggests that apposition of VDAC (voltage-dependent anion channel) and ANT (adenine nucleotide translocator) at contact sites between the mitochondrial outer and inner membranes together with matrix CypD (cyclophilin D) could be involved [35,36]. By using modulators of the PTPC formation, it was demonstrated that the *R(+) *enantiomer of C7a induced a pore with heterogenous composition, formed by ANT in higher concentration than with CypD molecules [21].

The oxidation of thiol groups in mitochondrial membrane proteins was a direct effect of the *R(+) *enantiomer of C7a, since generation of free radicals was not observed. Interestingly, the relative lipophilicity of the enantiomeric *R(+) *complex allows the preferential partitioning of the cyclopalladated compound into biological membranes and restricts its effects on critical thiol groups of membrane proteins, as suggested by the absence of oxidation of membrane lipids and GSH in isolated rat mitochondria incubated with the compound [14]. It remains to be determined whether or not GSH could act as a detoxifying agent for C7a in intact tumor cells.

The C7a complex caused mitochondrial swelling in intact tumor cells, as observed in transmission electron microscopy (TEM) images. Treatment of Jurkat cells with cisplatin also induced similar morphological alterations in mitochondria [37].

Marzo *et al*. [38,39] have shown that pro- and anti-apoptotic members of the Bcl-2 protein family may interact with the PTPC complex, suggesting that both effectors are not completely independent from each other, leading to mitochondrial membrane permeabilization. The insertion of Bax into mitochondrial membrane causes the release of cytochrome *c *and dissipation of mitochondrial membrane potential (ΔΨm). Citochrome *c *can interact with Apaf-1, and this complex in the presence of ATP, activates effector caspases and nucleases leading to the proteolysis of the cell and nuclear fragmentation, while the loss of ΔΨm corresponds to arrest in cellular energy production [reviewed in [40,41]]. The present results demonstrate that C7a induces the translocation of recombinant GFP-Bax from cytosol to mitochondria, suggesting that mitochondrial permeabilization caused by the cyclopalladated complex is associated with PTPC formation and Bax translocation to the mitochondrial membrane.

The mitochondrial membrane potential (ΔΨm) is a hallmark of the apoptotic cell death. During apoptosis, nuclear disintegration can be preceded by loss or entire dissipation of the ΔΨm [42], which may occur shortly before or simultaneously with Bax translocation to mitochondria membranes [20,24,43]. The mitochondrial permeabilization induced by C7a caused dissipation of ΔΨm in most of the tumor cell mitochondria, and this effect was completely abolished by previous incubation with DTT. In addition, it was also observed the inhibition of respiratory activity and decrease in energy production, as inferred from the decrease in the extracellular acidification [14] and ATP, respectively.

We also tested caspase activation in C7a-treated tumor cells. B16F10-Nex2 cells were incubated with C7a and caspase activation was measured in cell lysates showing a strong activation of caspases 2, 3 and 6. Caspases 3 and 6 were more activated in C7a- than in UV-treated cells, a classical apoptotic stimulus. Activation of caspase 3 was also observed by flow cytometry in C7a-treated tumor cells. Caspase activation and chromatin condensation presently shown and DNA degradation detected previously [14] indicated that C7a induced apoptotic cell death in tumor cells. The increase of caspase-8 activation suggests that C7a treatment of tumor cells may also affect a receptor-mediated reaction in addition to the intrinsic pathway of cell apoptosis.

The C7a complex induced an increase in cytoplasmic Ca^2+ ^probably also as an effect of Bax which modulates Ca^2+ ^concentration in mitochondria and the endoplasmic reticulum [44,45]. The involvement of Ca^+2 ^in the apoptotic cell death induced by C7a compound was corroborated by the inhibition of GFP-Bax translocation to mitochondria when cells were pre-incubated for 20 minutes with BAPTA-AM, an intracellular calcium chelator, before C7a addition.

Mitochondrial dysfunction has been implicated in apoptosis and necrosis. Necrosis has been regarded until recently as a passive and unregulated form of cell death, however, it is now recognized that, except under extreme conditions, this type of cell death is a well-regulated process and may be called necroptosis [46,47]. The protein kinase RIP1, associated with the necrotic cell death, can directly or indirectly induce mitochondrial permeability. Mitochondrial collapse activates various proteases (e.g., calpains, cathepsins) and phospholipases, and eventually leads to plasma membrane disruption, a hallmark of necrotic cell death [48]. Treatment with C7a did not induce plasma membrane disruption, as observed in light microscopy (Additional file 2, Figure S2). In addition, incubation of tumor cells with necrostatin, an inhibitor of RIP1 kinase [49], did not affect the cytotoxic effect of C7a (data not shown).

In a preclinical model of primary melanoma, C7a significantly delayed the subcutaneous murine melanoma B16F10-Nex2 development, prolonging mice survival [14]. In the present work, we demonstrated that C7a significantly reduced the number of lung nodules in endovenously-injected B16F10-Nex2 mice, suggesting that the cyclopalladated compound is effective also in controlling metastatic tumors. As demonstrated by Hebeler-Barbosa *et al*. [15], the C7a complex may be successfully used as an adjuvant in biochemotherapeutic protocols against murine melanoma.

The antitumor effect of C7a was also tested against human tumor cells, comparatively with cisplatin, the standard metallic complex used in chemotherapy. The IC_50 _values for the cyclopalladated compound, determined by 2 different methods, were 200 to 1.300 times lower than the cisplatin IC_50 _values for the same tumor cell line. In fact, even high doses (50 μM) of C7a were not cytotoxic to murine macrophages or human erythrocytes *in vitro *(data not shown). These *in vitro *toxicity results agree with *in vivo *assays, in which mice were treated with increasing doses of C7a for several weeks with no toxicity been observed in vital tissues [14]. As with murine melanoma, the C7a complex also affects the respiratory metabolism of human tumor cells, inhibiting the generation of protons in the respiratory chain, thus suggesting that the cyclopalladated compound may cause cell death by the same mechanism.

## Conclusions

Our results suggest that the C7a complex enters tumor cells passively, although it is not excluded the lateral interaction with a cell ligand. It induces the intrinsic apoptotic pathway, as demonstrated by the strong effect on mitochondria. C7a interacts with protein thiol groups in the mitochondrial membrane, induces Bax translocation to mitochondria, causes dissipation of ΔΨm, arrest of ATP production, inhibition of respiratory activity, cytosolic calcium increase and caspase/endonuclease activation.

The C7a complex was cytotoxic in murine and human tumor cells, including cisplatin-resistant lineages. In a preclinical model of melanoma, C7a was effective against primary and metastatic tumors, with low toxicity. The cyclopalladated C7a is therefore a promising tumor chemotherapeutic agent, to be used alone or in a combination therapy.

## List of abbreviations

ΔΨm: mitochondrial transmembrane potential; ATP: adenosine triphosphate; DTT: dithiothreitol; FCCP: carbonyl cyanide trifluoro-methoxyphenylhydrazone; GFP: green fluorescent protein; IC_50_: inhibitory concentration 50%; TEM: transmission electron microscopy; TMRE: tetramethylrhodamine ethyl ester.

## Competing interests

The authors declare that they have no competing interests.

## Authors' contributions

ACFC synthesized C7a complex; LSS performed the experiments for IC_50 _determination and together with ELS and JBP performed the experiments to determine the respiratory metabolism of tumor cells; FAS and FVP performed the *in vivo *experiments; FAS and JMJr participated in the determination of caspase activation; RMM determined ATP concentrations; FAS, ALM, PTM and AB performed all experiments for mitochondrial/cell parameters determination in intact tumor cells; TR and DPS performed the experiments with isolated rat liver mitochondria; LRT and SSS participated in the coordination of the study, analysis of data, and helped draft the manuscript; EGR conceived the study, participated in its design, coordination and analysis of data, and wrote the manuscript. All authors read and approved the final version of the manuscript.

## Pre-publication history

The pre-publication history for this paper can be accessed here:

http://www.biomedcentral.com/1471-2407/11/296/prepub

## Supplementary Material

Additional file 1 **Figure S1; Temperature does not affect C7a effect**. B16F10-Nex2 cells, 5 × 10^3 ^plated on 96-well plates, were treated with 1 μM C7a. The cells were incubated for 6 h at 37°C or 4°C, and viable cells were counted by Trypan blue exclusion. Incubation for 3 and 12 h showed the same result (not shown). Click here for file

Additional file 2 **Figure S2; Morphological alterations after C7a treatment**. B16F10-Nex2 cells (5 × 10^3^) were plated on 96-well plate and treated with 1 μM C7a. Alterations in cell morphology were observed after 5 **(B)**, 10 **(C) **and 25 **(D) **minutes. **(A) **Untreated tumor cells. Images were acquired in different areas of the cell culture. Magnification, 400 ×. Scale bars, 20 μm. Click here for file
