# Supplementary figures and images for: A cyclopalladated complex interacts with mitochondrial membrane thiol-groups and induces the apoptotic intrinsic pathway in murine and cisplatin-resistant human tumor cells
Source: BMC Cancer. 2011 Jul 14;11:296. doi: 10.1186/1471-2407-11-296 (PMC3156809; doi:10.1186/1471-2407-11-296)

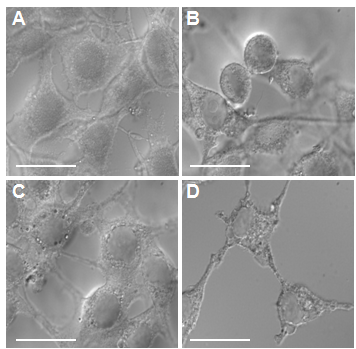

Supplement: Additional file 2 — Figure S2; Morphological alterations after C7a treatment. B16F10-Nex2 cells (5 × 10^3^) were plated on 96-well plate and treated with 1 μM C7a. Alterations in cell morphology were observed after 5 (B), 10 (C) and 25 (D) minutes. (A) Untreated tumor cells. Images were acquired in different areas of the cell culture. Magnification, 400 ×. Scale bars, 20 μm. [file 1471-2407-11-296-S2.TIFF]
